# The Prognostic Significance of Neutrophil-to-Lymphocyte Ratio in Head and Neck Cancer Patients Treated with Radiotherapy

**DOI:** 10.3390/jcm7120512

**Published:** 2018-12-03

**Authors:** Yeona Cho, Jun Won Kim, Hong In Yoon, Chang Geol Lee, Ki Chang Keum, Ik Jae Lee

**Affiliations:** 1 Department of Radiation Oncology, Gangnam Severance Hospital, Yonsei University College of Medicine, Seoul 06273, Korea; iamyona@yuhs.ac (Y.C.); junwon@yuhs.ac (J.W.K.); 2 Department of Radiation Oncology, Yonsei Cancer Center, Yonsei University College of Medicine, Seoul 03722, Korea; yhi0225@yuhs.ac (H.I.Y.); cglee1023@yuhs.ac (C.G.L.)

**Keywords:** head and neck cancer, radiotherapy, neutrophil/lymphocyte ratio, survival

## Abstract

Background: To investigate the prognostic value of pre-treatment neutrophil/lymphocyte ratio (NLR) in patients treated with definitive radiotherapy (RT) for head and neck cancer. Methods: We retrospectively analyzed 621 patients who received definitive RT for nasopharyngeal, oropharyngeal, hypopharyngeal, and laryngeal cancer. An NLR cut-off value of 2.7 was identified using a receiver operating characteristic curve analysis, with overall survival (OS) as an endpoint. Results: The 5-year progression-free survival (PFS) and OS for all patients were 62.3% and 72.1%, respectively. The patients with a high NLR (68%) had a significantly lower 5-year PFS and OS than their counterparts with a low NLR (32%) (PFS: 39.2% vs. 75.8%, *p* < 0.001; OS: 50.9% vs. 83.8%, *p* < 0.001). In a subgroup analysis according to primary site, a high NLR also correlated with a lower PFS and OS, except in oropharyngeal cancer, where a high NLR only exhibited a trend towards lower survival. In a multivariate analysis, a high NLR remained an independent prognostic factor for PFS and OS. Conclusion: Head and neck cancer tends to be more aggressive in patients with a high NLR, leading to a poorer outcome after RT. The optimal therapeutic approaches for these patients should be reevaluated, given the unfavorable prognosis.

## 1. Introduction

Currently, definitive radiotherapy (RT) is one of the main modalities used to treat locally advanced head and neck cancer. However, patients exhibit varying degrees of RT response and may develop recurrences even after a complete response. Although various clinical and molecular predictors of treatment outcomes after definitive RT for head and neck cancer have been investigated, no clear consensus regarding reliable predictive biomarkers has been reached.

Treatment outcomes are known to be affected by both tumor characteristics and host-related factors, including age, sex, and performance status. Recent reports have also described close associations of systemic inflammation with tumorigenesis and treatment outcomes [1,2], and several laboratory markers associated with systemic inflammatory processes, including albumin, hemoglobin, absolute white blood cell (WBC) count or WBC components, and platelet count, have been investigated as prognostic and predictive markers in various types of cancer [3,4]. Inflammation plays a key role in cancer physiology by promoting carcinogenesis, dedifferentiation, and primary tumor growth, and by stimulating tumor cell proliferation by inhibiting apoptosis and increasing mitotic rates [5]. 

Tumor–host interactions can induce systemic inflammatory responses that affect the numbers of circulating WBCs and the neutrophil/lymphocyte ratio (NLR) in certain types of cancers [6]. Normal NLR values are in the range of 0.78–3.53 in the general population [7]; high NLR values are associated with poor outcomes not only in cancer patients but also in patients with cardiovascular disease [8,9,10]. To date, some studies of head and neck cancer have suggested an association of high NLR with a poor prognosis. However, data regarding the prognostic significance of a high NLR are limited, especially among patients undergoing definitive RT [11,12,13]. The antitumor immune response is thought to be part of the ionizing radiation-induced tumor cell death process. Therefore, tumor shrinkage caused by the host immune response may be a direct effect of radiation [14,15]. Accordingly, we postulated that the host immune status, as reflected by the NLR, may predict recurrence after RT in head and neck cancer patients. This study aimed to evaluate the relationships of pretreatment NLR and other hematologic markers with tumor recurrence and survival in patients undergoing definitive RT for head and neck cancer.

## 2. Materials and Methods

### 2.1. Patient Selection and Treatment Protocols

This study was approved by the institutional review board of the Gangnam Severance Hospital (Protocol number: 3-2017-0387). We retrospectively reviewed the medical records of patients who underwent definitive RT with or without concurrent chemotherapy for cancers of the head and neck (including nasopharyngeal, oropharyngeal, hypopharyngeal, and laryngeal cancers) at our institution between 2006 and 2015. Patients who underwent surgery before or after RT, received RT of <30 Gy, had a distant metastasis at the initial diagnosis or previous history of other primary cancer, and whose pre-RT common blood test results were unavailable were excluded. The remaining 621 patients included in the analysis were staged according to the 7th edition of the TNM classification of the American Joint Committee on Cancer (AJCC). Human Papilloma Virus (HPV) infection status was evaluated in oropharyngeal cancer patients. To assess HPV status of each tumor, we used formalin-fixed, paraffin-embedded biopsy tissue to examine p16 expression, which is recognized as a surrogate marker for HPV infection in the oropharynx. Details of this process are described in a previous report of our institution [16]. 

Patients were treated with definitive RT alone, concurrent chemoradiotherapy (CCRT), or induction chemotherapy followed by CCRT. The choice of treatment was determined by the primary tumor and stage, risk factors, and/or the physicians’ discretion. External beam RT comprised either 3D-conformal RT or intensity-modulated RT (IMRT) and was administered 5 days per week in daily fractions of 1.8–2.5 Gy to yield total doses to the primary tumor of 66–75 Gy. 

Concurrent chemotherapy regimens included weekly cisplatin (DDP; 40 mg/m^2^); weekly 5-fluorouracil (5-FU) and cisplatin (FP; 750 mg/m^2^ and 20 mg/m^2^, respectively); and 5-FU, taxotere, and cisplatin (TPF; 750 mg/m^2^, 70 mg/m^2^, and 75 mg/m^2^, respectively) every 3 weeks. The anti-epidermal growth factor receptor mAb, cetuximab (Erbitux), was also used. The induction chemotherapy regimen consisted of FP every 3 weeks for 3 cycles or TPF every 3 weeks for 2 cycles.

### 2.2. Hematologic Markers

The patients’ blood counts were evaluated prior to performing diagnostic procedures or administering treatments. The WBC count, hemoglobin (Hb) level, absolute neutrophil count (ANC), absolute lymphocyte count (ALC), platelet count, and albumin level were recorded. A diagnosis of anemia was based on a hemoglobin level of <13 g/dL in men and <12 g/dL in women, and hypoalbuminemia was defined as a serum albumin level <3.5 g/dL. Onodera’s prognostic nutritional index (PNI) was calculated as 10 × Albumin + 0.005 × ALC. 

The NLR was calculated as the neutrophil count divided by the lymphocyte count, and the platelet/lymphocyte ratio (PLR) was calculated as the platelet count divided by the lymphocyte count. The optimum NLR cut-off values were identified via a receiver operating characteristic (ROC) curve analysis, using overall survival (OS) as an end point (Supplement Figure S1), and patients were categorized into high NLR (NLR ≥ 2.7) and low (NLR < 2.7) NLR groups. 

### 2.3. Outcome Assessment

All patients were followed up for 4–6 weeks after RT, and subsequently at 3-month intervals for the first and second years, 6-month intervals for the third year, and annually for the fourth and fifth years. Progression was defined as regrowth of the primary tumor or the involvement of cervical lymph node(s) (LN) or detection of any new lesion(s) in follow-up imaging studies. Progression-free survival (PFS) was defined as the interval between the date of initial treatment to the detection of first recurrence, death from any cause, or the last follow-up. OS was defined as the interval between the date of initial treatment and death from any cause or the last follow-up.

### 2.4. Statistical Analysis

Categorical data were analyzed using Fisher’s exact test or the χ^2^ test. Continuous data were compared between groups using the Mann–Whitney U test. The Kaplan–Meier method and log-rank test were used to estimate and compare the PFS and OS rates. Hazard ratios (HRs) were obtained using the cumulative survivor function and are reported with corresponding 95% confidence intervals (CIs). Univariate and multivariate analyses of factors related to OS and PFS were conducted using the Cox proportional hazards model, and multivariate analysis included all variables with *p* values < 0.05 in the univariate analysis. A *p* value < 0.05 was considered statistically significant. All analyses were performed using IBM SPSS, version 20.0 (SPSS, Chicago, IL, USA).

## 3. Results

### 3.1. Patient and Treatment Characteristics

Table 1 presents the demographic and treatment characteristics of the 621 included patients, of whom 425 (68.4%) and 196 (31.6%) were stratified into the low and high NLR groups, respectively. Laryngeal cancer was the most frequent type of primary cancer in the low NLR group, whereas nasopharyngeal cancer cases comprised the majority in the high NLR group. Patients with a high NLR tended to have a more advanced clinical T classification and higher frequency of LN metastasis and a significantly higher frequency of systemic chemotherapy (75.2% vs. 48.5%, *p* < 0.001) than those in the low NLR group. 

The groups did not differ significantly in terms of the use of IMRT. Of the 16 patients who did not complete the planned course of RT, four (2.0%) and 12 (2.8%) belonged to the high and low NLR groups, respectively (*p* = 0.787). However, more patients in the high NLR group received radiation doses of ≥70 Gy (equivalent dose in 2 Gy fractions, α/β = 10), and the overall duration of RT tended to be longer in this group. 

### 3.2. Hematologic Markers

Table 2 presents the values of the measured hematologic markers in all patients. The median baseline WBC count, ANC, and ALC were 6800/µL, 3900/µL, and 1810/µL, respectively, and the WBC and ANC values were significantly higher in the high NLR group. A higher proportion of patients with leukocytosis (WBC count ≥9000/µL) was also observed in the high NLR group (31.7% vs. 7.8% for low NLR, *p* < 0.001). The high NLR group also had higher platelet counts, which expectedly yielded a higher PLR, and was more likely to present with anemia and hypoalbuminemia at the time of diagnosis. Patients with a high NLR also had a significantly lower Onodera’s PNI (49.7 vs. 55 for low NLR, *p* < 0.001).

### 3.3. Survival Analysis

**Figure 1:**
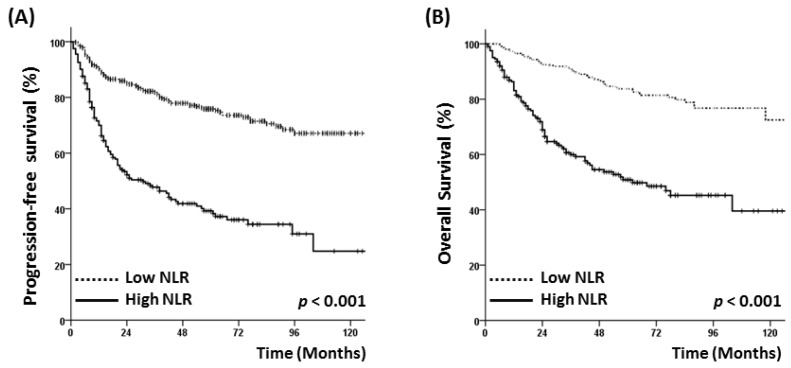
(**A**) Progression-free survival and (**B**) overall survival of patients according to neutrophil/lymphocyte ratio (NLR) status.

**Figure 2:**
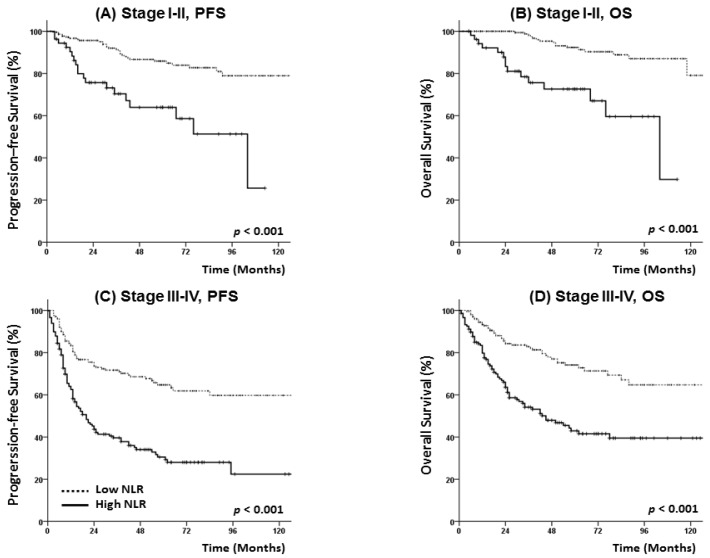
Progression-free survival and overall survival of patients with head and neck cancer at (**A**,**B**) stage I–II, and (**C**,**D**) stage III–IV.

The patients were followed up for a median of 39 (range, 2–130) months. During the follow-up period, 148 patients died and 156 experienced a recurrence. The 5-year PFS and OS rates for all patients were 63.8% and 72.9%, respectively, and both rates were significantly lower in the high NLR group than in the low NLR group (PFS: 39.2% vs. 75.8%, *p* < 0.001; OS: 50.9% vs. 83.8%, *p* < 0.001) (Figure 1). In survival analyses stratified by early- (stage I–II) or advanced-stage disease (stage III–IV), a high NLR remained significantly associated with a poor PFS and OS (Figure 2).

**Figure 3:**
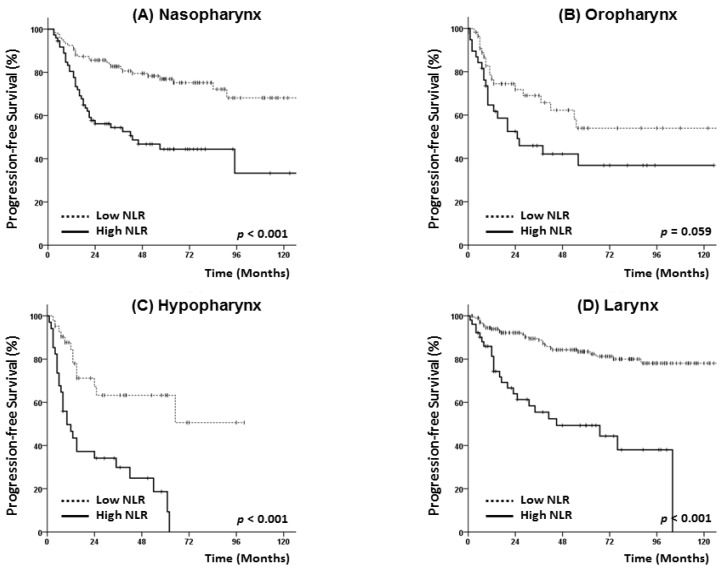
Subgroup analysis of progression-free survival according to primary tumor site: Nasopharynx (**A**), oropharynx (**B**), hypopharynx (**C**) and larynx (**D**).

**Figure 4:**
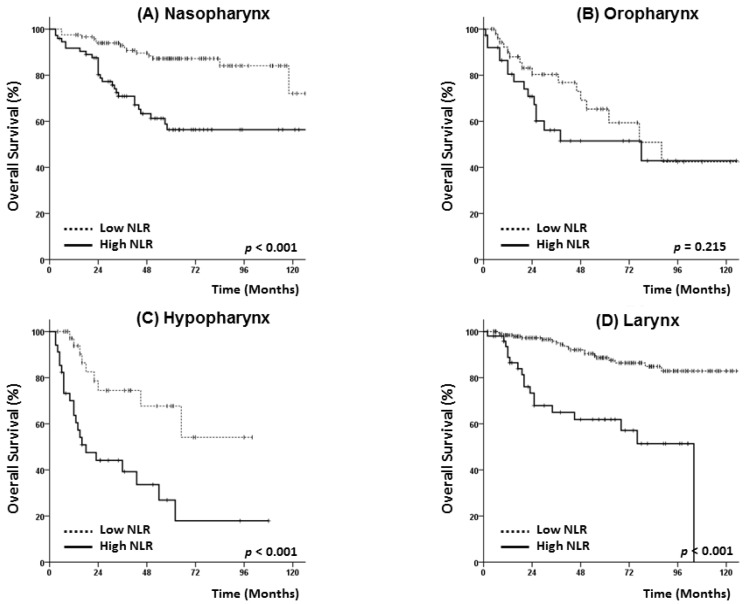
Subgroup analysis of overall survival according to primary tumor site: nasopharynx (**A**), oropharynx (**B**), hypopharynx (**C**) and larynx (**D**).

An additional subgroup analysis was performed after stratifying cases by the primary site (nasopharynx, oropharynx, hypopharynx, and larynx). As shown in Figure 3 and Figure 4, patients with nasopharyngeal, hypopharyngeal, and laryngeal cancer and the high NLR group had poorer PFS and OS rates. Among patients with oropharyngeal cancer, however, a high NLR status exhibited only borderline significance in terms of 5-year PFS (42.0% vs. 54.0%, *p* = 0.059) (Figure 3B) and only exhibited a trend with reduced OS (51.5% vs. 65.3%, *p* = 0.215) (Figure 4B). 

We also conducted additional analysis according to the treatment scheme. Both PFS and OS were significantly worse in high NLR patients receiving any type of treatment: Patients receiving RT alone, 5-year PFS 84.8% vs. 56.1%, *p* < 0.001 and OS 90.6% vs. 68.6%, *p* < 0.001; patients receiving CCRT, 5-year PFS 66.2% vs. 31.5%, *p* < 0.001 and OS 74.3% vs. 41.7%, *p* < 0.001; patients receiving induction chemotherapy + CCRT, 5-year PFS 68.1% vs. 40.7%, *p* < 0.001 and OS 78.9% vs. 55.2%, *p* < 0.001.

### 3.4. Analysis of Prognostic Factors

The results of univariate and multivariate analyses performed to identify prognostic factors for PFS and OS are shown in Table 3. The multivariate analysis revealed significant associations of a high NLR with poor PFS, an older age, and an advanced T classification. An elevated PLR was also found to be associated with a poor PFS. An older age, advanced T classification, and high NLR were also found to associate significantly with OS, and an elevated PLR exhibited a borderline significant association with a poor OS. Primary hypopharyngeal cancer was associated with both a poor PFS and a poor OS. A low albumin level (<3.3 g/dL) exhibited a negative trend with PFS, but not with OS. LN metastasis, overall stage, leukocytosis (WBC count ≥9000/µL), anemia, and Onodera’s PNI were not identified as prognostic factors for PFS or OS. 

### 3.5. p16 Status and Hematologic Markers

We also evaluated the relationship of the p16 status with the levels of various hematologic markers. This status was available for 38 of 94 patients with oropharyngeal cancer (40.4%). In total, 26 patients had p16-positive oropharyngeal cancer and 12 patients had p16-negative oropharyngeal cancer. Patients with a positive p16 status tended to have a lower NLR than those with a negative status (median NLR: 2.1 vs. 2.8, *p* = 0.103). In addition, the WBC count and ANC were lower in p16-positive patients than in their p16-negative counterparts, although these differences were not statistically significant (median WBC: 7100 vs. 8300/µL, *p* = 0.073; median ANC: 4000 vs. 5200/µL, *p* = 0.119) (Supplement Figure S2).

Among the 26 patients with p16-positive oropharyngeal cancer, PFS and OS were not different between the low and high NLR groups (5-year PFS 56.1% vs. 57.1%, *p* = 0.781 and 5-year OS 86.5% vs. 80.0%, *p* = 0.646). In patients with p16-negative oropharyngeal cancer (*n* = 12), PFS was significantly lower in the high NLR group than in the low NLR group (5-year PFS 0% vs. 100%, *p* = 0.009), while OS showed no difference, which may be due to the limited number of cases.

## 4. Discussion

During the past decade, various markers of systemic inflammation have been evaluated with the aims of refining patient stratification to treatment and predictions of survival. Of these markers, the NLR, which is derived from the ANC and ALC of a full blood count, is routinely available. Accordingly, in this study, we evaluated the significance of the pre-treatment NLR in patients who received RT for head and neck cancer and observed significant associations of a high NLR with disease recurrence and OS in the patient sample. 

As noted above, we stratified the patients into two groups according to NLR status and found that those with a high NLR had a more advanced clinical stage and therefore more frequently received concurrent chemotherapy with a higher total radiation dose and longer total duration of RT. Despite this more aggressive treatment, however, patients with a high NLR were more likely to experience unfavorable outcomes, and these results remained consistent regardless of disease stage. We further found that adverse hematologic features, such as an elevated WBC, ANC, and platelet count as well as anemia, hypoalbuminemia, and a low Onodera’s PNI, were more frequently observed in the high NLR group. These findings suggest that certain types of tumors elicit an enhanced systemic inflammatory response, which may reflect the aggressive nature of the tumor. 

We also performed a subgroup analysis according to the primary site of head and neck carcinoma and determined that a high NLR was significantly associated with a poorer PFS and OS among patients with nasopharyngeal, hypopharyngeal, and laryngeal cancers. However, among patients with oropharyngeal cancer, a high NLR was only borderline significant as a prognosticator of PFS and exhibited a trend with OS. Other studies of oropharyngeal cancer have identified NLR as a significant prognostic factor for disease control, and most have used a relatively higher NLR cut-off value (≥5) [17,18] than those used for other primary sites in the head and neck [19,20]. Additionally, some studies of oropharyngeal cancer have identified the prognostic significance of circulating neutrophil and lymphocyte counts only for recurrence-free survival (RFS), but not for OS [21]. 

Our above findings may be attributable to the limited number of oropharyngeal cancer patients in our study (*n* = 94) or to the effects of HPV infection. Previous studies suggested that HPV infection might affect the distribution of WBC components and alter inflammatory responses in patients with oropharyngeal cancer [11]. Huang et al. reported that HPV-positive patients had lower levels of circulating neutrophil and monocyte counts when compared to their HPV-negative counterparts, despite similar levels of lymphocyte counts [21]. In the HPV-positive cohort, a high neutrophil or monocyte count was found to correlate with reductions in OS and RFS, whereas in the HPV-negative cohort, the neutrophil and lymphocyte counts were not predictive of either survival parameter. Another study also reported a significantly lower NLR among HPV-positive patients than among their HPV-negative counterparts. Given these findings, we suggest that oropharyngeal cancer may exhibit behaviors or inflammatory responses that are distinct from those of head and neck cancers at other primary sites. Although our study did not reveal significant differences in inflammatory markers among oropharyngeal cancer patients according to p16 status, HPV infection might have altered the WBC distribution and affected disease control and OS. 

Some scientists have recommended that more accurate diagnostic tools are needed for risk stratification according to HPV infection. Previous reports have suggested that HPV specific tests such as DNA in situ hybridization and polymerase chain reaction should be used to confirm HPV status, although p16 protein over-expression is very sensitive to the presence of transcriptionally-active HPV, correlates strongly with patient outcomes, is widely available, and is easy to interpret [22]. Gupta et al. also suggested that circulating HPV16 DNA in the plasma may be a clinically useful biomarker [23]. These biomarkers can be measured using a simple blood test, and the level of ctDNA is predictive of an early treatment response. If used in combination with inflammatory markers, this technique will be helpful in predicting a patient′s prognosis and determining an appropriate treatment plan.

As noted, NLR is an easily and routinely determined biomarker of systemic inflammation, and in our study population a high NLR correlated strongly and independently with a poorer PFS and OS. Previous studies have also identified a high NLR as an independent prognostic factor for many other types of cancers, including colorectal cancer, renal cell cancer, pancreatic cancer, and head and neck squamous cell carcinoma [2,24,25,26,27,28]. Although the tumorigenic mechanism underlying this relationship with the NLR has not been clearly elucidated, it appears likely that increased levels of several inflammatory cytokines contribute to a microenvironment that promotes carcinogenesis and tumor progression [29]. Several growth factors, including epidermal growth factor, vascular endothelial growth factor, and transforming growth factor-α, also contribute to the creation of microenvironments supportive of angiogenesis and tumor proliferation [30]. Our results are therefore consistent with the concept that a high NLR contributes to poor disease control by suppressing the cytolytic activities of activated effector T cells and the peritumoral infiltration of immuno-suppressive cells such as macrophages [31,32].

We further demonstrated that a higher PLR was associated with PFS in our multivariate analysis. Again, the significance of interactions between platelets and the tumor microenvironment remains somewhat unclear. The platelet count provides an additional index of systemic inflammation elicited by the tumor and degranulation. This inflammation, together with the consequent release of platelet-derived proangiogenic mediators within the tumor microvasculature, may also serve as an important determinant of tumor growth [33,34,35]. 

This study had a few limitations. First, the study had a retrospective design and included various primary sites of head and neck cancer. Additionally, patients in the high NLR group tended to have more advanced-stage disease. However, the NLR remained a significant prognostic factor for survival in the multivariate analysis, as well as in an additional analysis according to disease stage. Second, the selection of treatment modalities and regimens was heterogeneous and was determined in accordance with the primary site and physicians’ discretion. Third, we did not explore the association of LN metastasis with patient outcomes because of the use of clinical stage and likelihood of upstaging for LNs. Therefore, our findings should be interpreted cautiously. Nevertheless, this is one of the largest studies to evaluate the prognostic significance of systemic inflammation in patients who underwent RT for head and neck cancer, and we have revealed potential differences in the patients’ characteristics and outcomes according to their NLR status. In addition, we observed different associations of the NLR status among patients with oropharyngeal cancer versus those with other head and neck cancers, which underscores the need for further studies of the relationship between HPV infection and the NLR. 

In conclusion, the results of our large population study validate the suggested association of a high NLR with poorer outcomes after adjusting for potential confounding factors. Although further studies of the biological mechanisms underlying the relationship between inflammation and aggressiveness are needed, our results suggest that a classification system based on pretreatment hematologic markers could identify patients with a high risk of recurrence and poor survival.

## Figures and Tables

**Table 1:** Patient characteristics.

		Total		Low NLR Group		High NLR Group		
Characteristics		*N* = 621	(%)	*N* = 425	(%)	*N* = 196	(%)	*p* Value
Age	Median	60		60		60		0.166
	(Range)	(18–94)		(18–89)		(18–94)		
Sex	Male	514	(82.8)	365	(85.9)	149	(76.0)	0.002
	Female	107	(17.2	60	(14.1)	47	(24.0)	
Primary site	Nasopharynx	192	(30.9)	119	(28.0)	73	(37.2)	<0.001
	Oropharynx	94	(15.1)	56	(13.2)	38	(19.4)	
	Hypopharynx	76	(12.2)	42	(9.9)	34	(17.3)	
	Larynx	259	(41.7)	208	(48.9)	51	(26.0)	
T classification	T1	259	(41.7)	206	(48.5)	53	(27.0)	<0.001
	T2	145	(23.3)	97	(22.8)	48	(24.5)	
	T3	94	(15.1)	52	(12.2)	42	(21.4)	
	T4	123	(19.8)	70	(16.5)	53	(27.0)	
N classification	N0	294	(47.3)	236	(55.5)	58	(29.6)	<0.001
	N1	87	(14.0)	56	(13.2)	31	(15.8)	
	N2	219	(35.3)	124	(29.2)	95	(48.5)	
	N3	21	(3.4)	9	(2.1)	12	(6.1)	
Overall stage	I	188	(30.3)	165	(38.8)	23	(11.7)	<0.001
	II	89	(14.3)	58	(13.6)	31	(15.8)	
	III	118	(19.0)	76	(17.9)	42	(21.4)	
	IVA	204	(32.9)	116	(27.3)	88	(44.9)	
	IVB	22	(3.5)	10	(2.4)	12	(6.1)	
p16	UE ^b^	56	(59.6)	31	(55.4)	25	(65.8)	0.529
(in oropharynx)	negative	12	(1.9)	6	(10.7)	6	(15.8)	
	positive	26	(4.2)	19	(33.9)	7	(18.4)	
Treatment	RT alone	270	(43.5)	221	(52.0)	49	(25.0)	<0.001
	CCRT ^c^	234	(37.7)	130	(30.6)	104	(53.1)	
	Induction + CCRT	117	(18.8)	74	(17.4)	43	(21.9)	
RT modality	3D-CRT ^d^	220	(35.4)	156	(36.7)	64	(32.7)	0.326
	IMRT ^e^	401	(64.6)	269	(63.3)	132	(67.3)	
RT duration	Median	46		45		47		<0.001
(days)	(Range)	(23–99)		(23–99)		(31–97)		
Total dose	<70	385	(61.0)	277	(64.6)	108	(53.5)	0.016
(EQD2_Gy_^a^, α/β = 10)	≥70	236	(37.4)	148	(34.5)	88	(43.6)	

Abbreviations: ^a^ Equivalent dose in 2 Gy fractions, ^b^ unevaluable, ^c^ concurrent chemoradiotherapy, ^d^ 3-dimensional conformal radiotherapy, ^e^ intensity modulated radiotherapy.

**Table 2:** Hematologic markers.

		Total		low NLR Group		High NLR Group		
Characteristic		*N* = 621	(%)	*N* = 425	(%)	*N* = 196	(%)	*p* Value
WBC	Median	6800		6300		7700		<0.001
(cells/μL)	(range)	(2000–21,100)		(2000–10,700)		(3700–21,100)		
ANC ^a^	Median	3900		3460		5520		<0.001
(cells/μL)	(range)	(560–18,310)		(940–6720)		(560–18,300)		
ALC ^b^	Median	1810		2040		1320		<0.001
(cells/ μL)	(range)	(160–3700)		(700–3700)		(160–2970)		
Platelet	Median	237		232		250		<0.001
(×10^3^ cells/μL)	(range)	(14–600)		(14–517)		(40–600)		
PLR ^c^	Median	131		116		194		<0.001
	(range)	(20–1733)		20–269		(26–1733)		
Hemoglobin	Median	14		14.1		13.5		0.01
(mg/dL)	(range)	(51–15.1)		(5.1–17.4)		(8.3–51.1)		
Anemia	No	497	(80.0)	359	(84.5)	138	(70.4)	<0.001
	Yes	124	(20.0)	66	(15.5)	58	(29.6)	
Albumin	Median	4.4		4.4		4.3		<0.001
(g/dL)	(range)	(2.5–5.2)		(2.7–5.2)		(2.5–5.1)		
Hypoalbuminemia	UE ^e^	69	(11.1)	49	(11.5)	20	(10.2)	0.02
(<3.3 g/dL)	No	539	(86.8)	371	(87.3)	168	(85.7)	
	Yes	13	(2.1)	5	(1.2)	8	(4.1)	
Onodera’s PNI ^d^	Median	53.1		55		49.7		<0.001
	(range)	(27.8–67.6)		(32.8–67.6)		(27.8–61.7)		

Abbreviations: ^a^ Absolute neutrophil count, ^b^ absolute lymphocyte count, ^c^ platelet/ lymphocyte ratio, ^d^ prognostic nutritional index, ^e^ unevaluable.

**Table 3:** Prognostic factors for progression-free survival and overall survival.

		Progression-Free Survival						Overall Survival					
Variable		Univariate Analysis			Multivariate Analysis			Univariate Analysis			Multivariate Analysis		
		HR	95%CI	*p*	HR	95%CI	*p*	HR	95%CI	*p*	HR	95%CI	*p*
Age	<60	1			1			1			1		
	≥60	1.61	1.22–2.12	0.001	1.54	1.08–2.19	0.017	2.40	1.71–3.37	<0.001	2.43	1.57–3.75	<0.001
Sex	Male	1						1					
	Female	0.97	0.68–1.38	0.865				0.99	0.66–1.51	0.981			
Primary site	Nasopharynx	1			1			1			1		
	Oropharynx	1.78	1.21–2.62	0.003	1.04	0.67–1.61	0.859	2.29	1.44–3.62	<0.001	1.19	0.68–2.09	0.54
	Hypophrynx	2.46	1.66–3.66	<0.001	1.88	1.17–3.01	0.009	3.41	2.13–5.44	<0.001	2.01	1.13–3.55	0.017
	Larynx	0.63	0.44–0.91	0.012	1.07	0.65–1.76	0.786	0.74	0.48–1.15	0.181	0.97	0.49–1.90	0.924
T classification	T1, 2	1			1			1			1		
	T3, 4	3.05	2.32–4.00	<0.001	2.03	1.37–3.00	<0.001	3.53	2.54–4.90	<0.001	2.39	1.48–3.86	<0.001
N classification	N0	1			1			1			1		
	N1-3	2.78	2.06–3.77	<0.001	1.37	0.81–2.32	0.237	2.52	1.76–3.60	<0.001	1.11	0.60–2.04	0.749
Overall stage	I-II	1			1			1			1		
	III-IVB	3.48	2.52–4.81	<0.001	1.44	0.78-2.67	0.244	3.76	2.53–5.60	<0.001	1.58	0.71–3.48	0.26
Treatment	RT alone	1			1			1			1		
	CCRT ^e^	2.83	2.06–3.90	<0.001	1.11	0.63–1.96	0.723	2.65	1.81–3.88	<0.001	1.02	0.53–1.98	0.951
	Induction + CCRT	2.09	1.42–3.07	<0.001	0.77	0.41–1.46	0.425	1.98	1.25–3.13	0.003	0.85	0.38–1.64	0.519
RT modality	3D CRT ^f^	1						1					
	IMRT ^g^	1.26	0.95–1.68	0.107				1.19	0.86–1.66	0.298			
Anemia	No	1			1			1			1		
	Yes	2.25	1.69–3.00	<0.001	1.19	0.81–1.74	0.374	0.52	0.37–0.72	<0.001	0.96	0.63–1.45	0.829
WBC (cells/μL)	<9000	1			1			1			1		
(cells/μL)	≥9000	1.81	1.30–2.52	<0.001	0.93	0.61–1.43	0.748	1.62	1.08–2.42	0.021	0.73	0.43–1.23	0.233
ANC ^a^	<4000	1			1			1			1		
(cells/μL)	≥4000	1.81	1.38–2.39	<0.001	0.75	0.51–1.10	0.142	2.02	1.45–2.81	<0.001	0.79	0.50–1.27	0.333
ALC ^b^	<2000	1			1			1			1		
(cells/μL)	≥2000	0.61	0.45–0.82	0.001	1.10	0.72–1.68	0.672	0.55	0.38–0.79	0.001	1.10	0.64–1.89	0.72
Platelet	<230	1						1					
(10^3^ cells/μL)	≥230	0.98	0.74–1.28	0.857				1.04	0.75–1.44	0.825			
NLR	<2.7	1			1			1			1		
	≥2.7	3.39	2.58–4.46	<0.001	4.10	2.66–6.34	<0.001	3.86	2.78–5.35	<0.001	4.63	2.69–7.94	<0.001
PLR ^c^	<150	1			1			1			1		
	≥150	1.79	1.36–2.36	0.001	1.57	1.20–2.06	0.03	1.96	1.42–2.74	0.001	1.24	0.96–2.40	0.096
Hypoalbuminemia	No	1			1			1			1		
	Yes	4.18	2.20–7.93	<0.001	1.92	0.95–3.85	0.068	0.44	0.3–0.66	<0.001	1.54	0.69–3.42	0.288
Onodera’s PNI ^d^	<50	1			1			1			1		
	≥50	0.48	0.36–0.65	<0.001	0.80	0.53–1.23	0.316	0.44	0.31–0.63	<0.001	0.74	0.45–1.24	0.259

Abbreviations: ^a^ Absolute neutrophil count, ^b^ absolute lymphocyte count, ^c^ platelet/ lymphocyte ratio, ^d^ prognostic nutritional index, ^e^ concurrent chemoradiotherapy, ^f^ 3-dimensional conformal radiotherapy, ^g^ intensity modulated radiotherapy.

## Supplementary Materials

The following are available online at http://www.mdpi.com/2077-0383/7/12/512/s1, Figure S1: The receiver operating characteristic (ROC) curve analysis for neutrophil/lymphocyte ratio (A) using overall survival OS as an end point. Figure S2. The white blood cell count (A) and neutrophil/lymphocyte ratio (B) in p16 negative and p16 positive patients.
