# Supplementary figures and images for: The Prognostic Significance of Neutrophil-to-Lymphocyte Ratio in Head and Neck Cancer Patients Treated with Radiotherapy
Source: J Clin Med. 2018 Dec 3;7(12):512. doi: 10.3390/jcm7120512 (PMC6306798; doi:10.3390/jcm7120512)

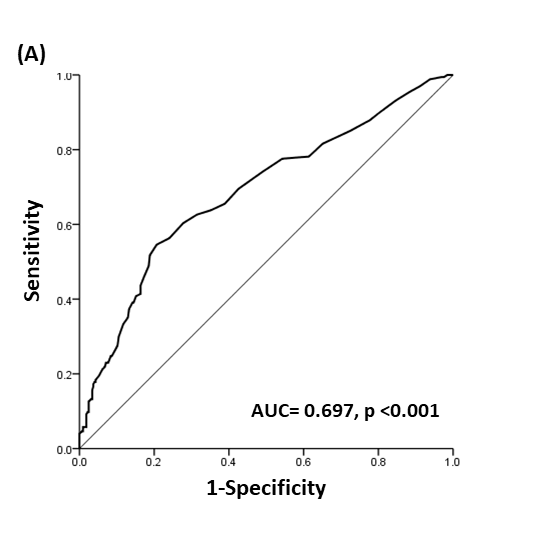

Supplement: Supplementary file 1 [file jcm-07-00512-s001.zip › Supplement figure 1.tif]

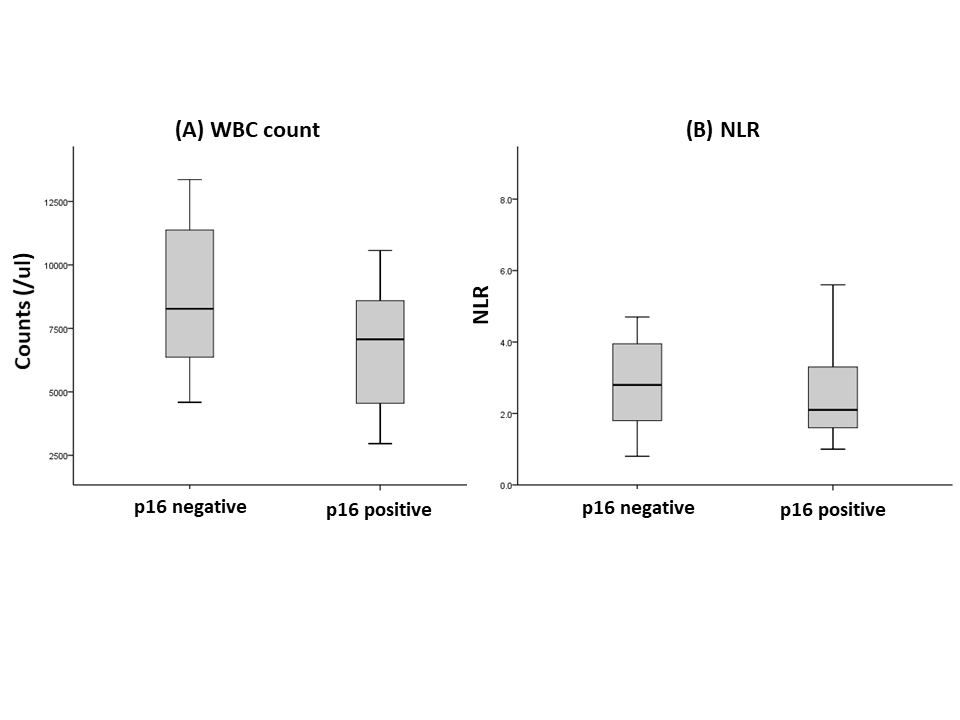

Supplement: Supplementary file 1 [file jcm-07-00512-s001.zip › Supplement figure 2.tif]
